# Profile of gym-goers who do not use performance-enhancement substances

**DOI:** 10.3389/fpsyg.2024.1357566

**Published:** 2024-05-30

**Authors:** Ana Sofia R. Tavares, Elisabete Carolino, Pedro Teques, Luis Calmeiro, Sidonio Serpa

**Affiliations:** ^1^H&TRC- Health & Technology Research Center, ESTeSL – Escola Superior de Tecnologia da Saúde, Instituto Politécnico de Lisboa, Lisbon, Portugal; ^2^N2i, Polytechnic Institute of Maia, Maia, Portugal; ^3^Research Center in Sports Sciences, Health Sciences and Human Development (CIDESD), Vila Real, Portugal; ^4^National Institute of Education, Physical Education and Sports Science, Nanyang Technological University, Singapore, Singapore; ^5^Institute of Environmental Health, Faculty of Medicine, University of Lisbon, Lisbon, Portugal; ^6^Research Center in Sport, Physical Education, Exercise and Health (CIDEFES), Universidade Lusofona, Lisbon, Portugal

**Keywords:** gym-goers, characteristic, behavior intentions, decision trees, doping, substance use

## Abstract

**Introduction:**

Currently the use of prohibited performance-enhancing substances (PES) in fitness and gym settings is a public health concern as adverse health consequences are emerging. Understanding the characteristics of gym-goers who do not use these substances could lead to an important complement to the ongoing research about risk factors for PES use. The aim of this study was to identify the profile of PES non-use in gym-goers.

**Methods:**

In total, 453 gym-goers (mean age = 35.64 years; SD = 13.08 – measure of central tendency location and measure of absolute dispersion, respectively) completed an online survey assessing sociodemographic factors, exercise characteristics, gym modalities, peers, social influence, attitudes, subjective norms, beliefs, intentions, and self-reported use of PES.

**Results:**

Decision Trees showed that being a woman, training less frequently, not practicing bodybuilding and having a negative intention to consume PES were identified as characteristics of non-users of PES.

**Discussion:**

These results may support evidence-based anti-doping interventions to prevent abusive use of PES in the fitness context.

## 1 Introduction

The research on psychosocial factors associated with the consumption of prohibited performance-enhancing substances (PES) in sports, physical activity, and fitness has increased over the past 20 years (e.g., Wiefferink et al., 2008; Morente-Sánchez and Zabala, 2013; Ntoumanis et al., 2014; Tavares et al., 2019a). Researchers’ interest in this area may be attributed to the prevalence rates of prohibited substance [e.g., anabolic-androgenic steroids (AAS), stimulants, erythropoietin (EPO), human growth hormone, and diuretics] use, which was shown to be as high as 73% in competitive sports (Gleaves et al., 2021), and 70% in the fitness context (Tavares et al., 2019a). Moreover, the media impact of cases involving performance-enhancing substance use in major global sports competitions, such as the Olympic Games (Associated Press/NBC, 2022) or the FIFA World Cup (Bubel, 2023), further contributes to the attention this topic has received.

While specific regulations and a publication of a list of prohibited substances by the World Anti-Doping Agency (WADA) exist for professional sports contexts to inform and control behavior associated with PES use, only a few countries have legal procedures for controlling fitness participants’ substance use (Thualagant, 2012). This fact should be a concern for these substances have long-term physical and psychological consequences on individuals’ health (Pope et al., 2014). To address this concern, this article will focus on the identification of the profile of gym-goers who do not engage in this risky behavior.

Researchers have been trying to understand and evaluate the psychological determinants of PES use by fitness participants (Elbe and Barkoukis, 2017). Studies have focused on attaining knowledge of predisposing factors influencing the decision to consume PES to influence consumption behavior and prevent such acts (Petróczi and Aidman, 2008). This is evident in the multitude of variables analyzed to assess determinants associated with PES use in fitness, such as participants’ attitudes and judgments, beliefs about the outcomes or consequences, and social influences on engaging in PES use (e.g., Allahverdipour et al., 2012; Tavares et al., 2020a).

Although scientific evidence identifies a set of psychological and social determinants associated with PES use, including intentions, attitudes, and beliefs (e.g., Wiefferink et al., 2008; Tavares et al., 2020a), it is important to examine the reasons for non-use, that is, to identify the characteristics of those gym-goers who do not use PES. The identification of the profile of these individuals will help distinguish risk characteristics prone for PES use from preventive ones. Emphasizing the promotion of the latter factors rather than focusing on penalizing the use of PES by gym-goers might be an alternative strategy to promote a safe engagement in gym activities (Henning and Andreasson, 2022). The potential negative health impact of PES use and its increased prevalence in gym-goers (Lazuras et al., 2017; Tavares et al., 2020b), focusing on individuals’ capacity to develop health assets that empower them to engage in health-protective behaviors (Morgan and Ziglio, 2007) requires an understanding of the profile of those who do not consider or refuse PES use.

Therefore, understanding the motivations of those gym-goers who reject PES, despite social pressure to consume (Copeland and Potwarka, 2016), may involve identifying a set of beliefs, attitudes, norms, and intentions, concerning substance use (Barkoukis et al., 2013). Following recent research that explained these cognitive aspects associated with PES use in the fitness context (Tavares et al., 2020a), the present study adopts the main concepts of the Theory of Planned Behavior (TPB; Ajzen and Fishbein, 1980; Ajzen, 1991) to explain the determinants of non-use of PES.

Over the past years, researchers have found that athletes’ substance use is determined by their intentions to engage in behaviors aimed at improving performance (e.g., Barkoukis et al., 2013; Ntoumanis et al., 2014). According to researchers who have conducted studies based on TPB (e.g., Chan et al., 2015a; Kirby et al., 2016), these intentions for PES use, in turn, are determined by three main social cognitive factors. First, they are influenced by athletes’ attitudes toward substance use. Attitudes toward PES use depends on the beliefs about the outcomes of the behavior and the judgments about its personal consequences, that is, the costs and benefits associated with substance use, as well as the emotional value athletes attach to these consequences. Second, intentions are determined by the subjective norms related to substance use. Subjective norms represent individuals’ perceptions of what their significant others believe they should do (normative beliefs) and whether they are motivated to act accordingly to those beliefs (motivation to comply) (Albarracín et al., 2001). Lastly, the perceived behavioral control (PBC) over PES consumption refers to one’s beliefs about the barriers and the perceived power one exerts over those barriers. In addition to its influence on intentions, PBC also directly influences behaviors when individuals have incomplete control over the behavior (Martinez and Lewis, 2016).

The TPB framework has also been utilized to explain PES use in gym-goers. Allahverdipour et al. (2012) were able to predict 63% of the intention to use PES based on PBC, subjective norm and attitudes. However, intention was the only variable that predicted self-reported PES use. Based on TPB, Tavares et al. (2019b) conducted a validation study on the Questionnaire of Attitudes toward Doping in Fitness (QAD-FIT). The emerging factor structure resulted in an instrument that assesses four dimensions related to the psychosocial aspects of PES use within the context of fitness. These dimensions consisted of attitudes, subjective norms, beliefs, and intentions regarding PES use. Subjective norms were the strongest predictor of intentions, followed by beliefs and attitudes. The three variables predicted 75% of intentions to use PES, which in turn predicted 44% of PES use. Subjective norms as the strongest predictor supports the important role of perceived social norms; indeed, first-hand accounts of peers have been identified as a major source of information for anabolic steroids users in the gym population (Kimergård, 2015).

Some research has explored the reasons behind not using PES. In a large study that encompassed five European countries, the most frequent reasons for young adults not to engage in this behavior were worry about the negative health impact, not feeling the need to use them, and a desire to find out how much individuals can achieve on their own. Not being able to afford these substances or not having access to them were not generally important reasons for non-use (Lazuras et al., 2017). These results reinforce the need for educational practices that emphasize the development of individuals’ empowerment.

In addition to these psychosocial determinants other demographic variables are likely to influence behavior. For example, being male, having lower educational qualifications, being unemployed, high training frequency and practicing bodybuilding were more likely to engage in PES compared to their counterparts (Tavares et al., 2022). A global profile of PES users further included “a desire to increase muscle mass, shape their body, and improve physical condition; being advised by friends, training colleagues and coaches or on the Internet” (p.10). Moreover, AAS male users demonstrated greater likelihood to meet criteria for substance dependence disorder, and reported having an anxiety disorder, frequent recent use of cocaine and a history of sexual abuse (Ip et al., 2011) when compared to their male non-user counterparts.

Considering the evidence that suggests the involvement of cognitive determinants in PES use (e.g., Wiefferink et al., 2008; Tavares et al., 2019a,2020a), it is important to understand how these determinants can constitute protective factors in individuals who do not report use of PES. Therefore, the present study’s main objective is to identify the profile of the non-use of PES of gym-goers. Accordingly, it is expected that unfavorable attitude toward substance consumption, the absence of a subjective norm that emphasizes consumption and, finally, unfavorable beliefs about the outcomes of PES consumption are associated with reports of non-use of PES. Additionally, it is also hypothesized that intentions toward PES use is negatively associated with self-reports of PES non-use. Finally, it is hypothesized that females and those who practice non-strength-based activities are more likely to report non-use of PES.

## 2 Materials and methods

### 2.1 Participants

Participants were 453 Portuguese gym-goers with ages ranging from 16 to 79 years (mean: 35.64; SD: 13.08), of both female (277; 61.1%) and male genders (175; 38.6%), and one did not indicate their gender (0.3%). Inclusion criteria included: practicing any gym activity, being older than 16 years of age, having capacity to read, and access to a smartphone, tablet, or PC to respond the online survey. To identify an appropriate sample size, we used an *a priori* sample size calculator (Soper, 2017). A sample of 434 participants were required to achieve a power of 0.90, and an anticipated effect size of 0.20 (meaning that the study has a 90% chance of detecting an association with the specified effect of 20%). The significance level was 0.05.

### 2.2 Measures

#### 2.2.1 Self-reported use of PES

After responding “yes” or “no” to the question “As part of your practice, have you ever taken performance-enhancing substances?” two groups were formed according to their answers (users and non-users). We considered the WADA Prohibited List to define PES which excludes dietary supplements and vitamins (Supplementary Appendix A).

#### 2.2.2 Questionnaire of attitudes toward doping in fitness

The QAD-FIT is composed by 16 items grouped into four dimensions based on the TPB: attitudes (five items; e.g., “Selling PES should be punished”), subjective norms (three items; e.g., “I would take PES, if most people I know approved of it”), beliefs (three items; e.g., “Performance enhancing substances help to improve physical abilities”), and intention (five items; e.g.; “I would take PES to achieve my goals in the practice of physical activity”). Answers to the questionnaire were given on a seven-point Likert-type scale where 1 corresponded to “totally disagree” and 7 to “totally agree.” The total composite reliability (CR) for QAD-FIT was 0.85, with values of 0.74 for beliefs, 0.84 for attitudes, 0.86 for subjective norms, and 0.97 for intentions (Tavares et al., 2019b).

### 2.3 Procedures

Institutional e-mail and Facebook accounts of Lisbon fitness centers were used to advertise the research and recruit participants, between October and November 2017. Those who accepted to participate accessed the survey web link where a participant information and informed consent page was presented. Here, information regarding the study was given as well as the explanation concerning how anonymity and confidentiality were guaranteed. It was not possible for data to be traced back to individual participants or their Internet providers. Moreover, encryption was performed during data transfer. Demographic data, self-reported use of PES (doping behavior), and attitudes, subjective norms, beliefs, and PES use intention were collected using a web-based survey administered via REDCap software (Version 5.11.4, Vanderbilt University). Fifteen minutes was the approximate time needed to complete the questionnaire. Ethical approval for the study was obtained from the Ethical Committee of the University of Lisbon, Faculty of Human Kinetics (study protocol no. 38/2017).

### 2.4 Data analysis

Data were analyzed using SPSS V27.0 statistical software for Windows. Results were considered statistically significant at a significance level of 5%. To identify the profile of non-PES users, Decision Trees were used, which are a method widely used in classifying and identifying profiles, in machine-learning and data mining. The method used to create the nodes of the decision tree was CHAID, because in addition to qualitative variables the database also included quantitative variables. For the decision tree models, split-sample validation was considered, using random assignment, where 50% of the data were used for the training phase and the remaining 50% were used for the testing phase. The results presented are those of the test phase. To understand the relationship between beliefs, subjective norms, attitudes and intention, multiple linear regression analysis was used. The models obtained obey the Gauss–Markov conditions (residuals with zero mean, constant variance, and Normal distribution) and do not present multicollinearity between the independent variables, evaluated through the tolerance value (whereby values <0.1 indicate the existence of multicollinearity) and/or the VIF (values >10 suggest multicollinearity) (Pestana and Gageiro, 2014; Marôco, 2021).

## 3 Results

### 3.1 Sociodemographic characteristics

**FIGURE 1:**
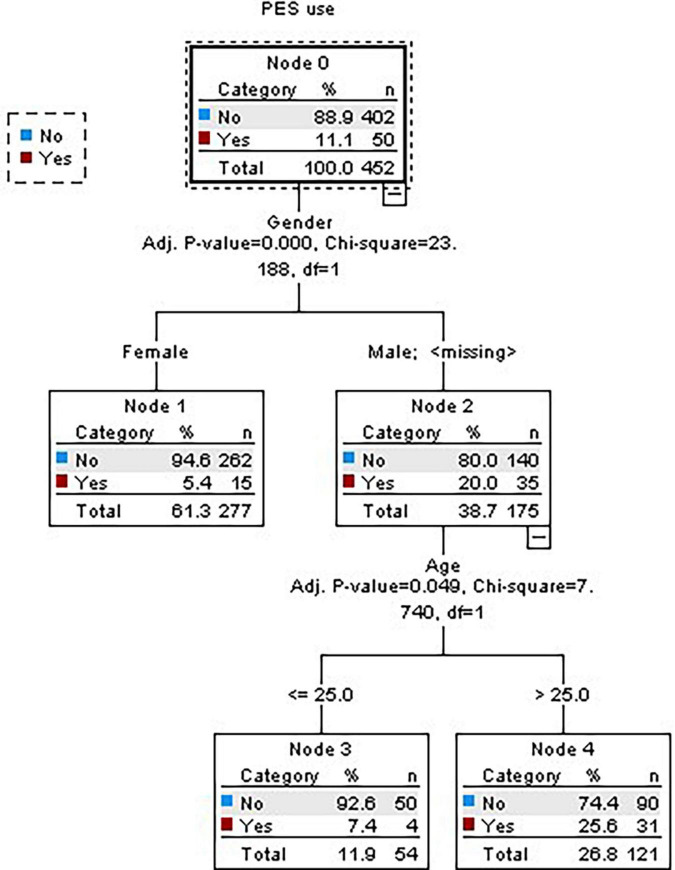
Decision tree for sociodemographic characteristics.

The use of PES was reported by 50 participants (11.1% of the 453 participants). Considering sociodemographic characteristics (gender, age, education, marital status, and occupation), only gender and age were significant, verifying that 94.5% of women do not consume PES. As far as men are concerned, 80% do not consume and those under 25 years old, 92.6% do not consume (Figure 1).

### 3.2 Activities, training frequency, and training time

**FIGURE 2:**
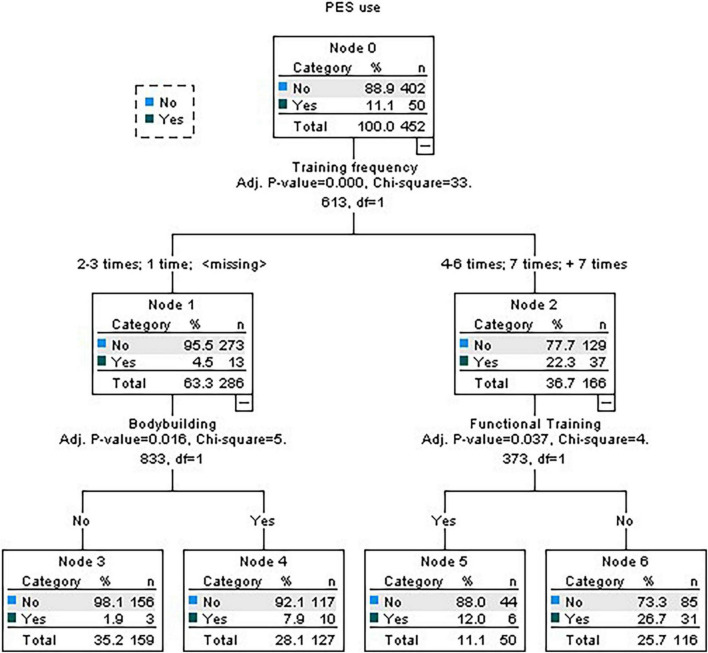
Decision tree for activities practiced, training frequency, and training time.

The activities in which the participants were involved were cardio fitness (57%), recreational bodybuilding (56.5%), stretching (27.8%), and localized gymnastics (27.2%). The frequency of training, bodybuilding and functional training were significant. Of those who have a lower training frequency (1, 2, or 3 times a week), 95.5% do not consume PES. Among these, 98.1% do not take PES and do not practice bodybuilding. Regarding those who have a higher training frequency (4 to 6 times, 7 or more times) and who practice functional training, 88% do not consume PES (Figure 2).

### 3.3 Psychosocial determinants

**FIGURE 3:**
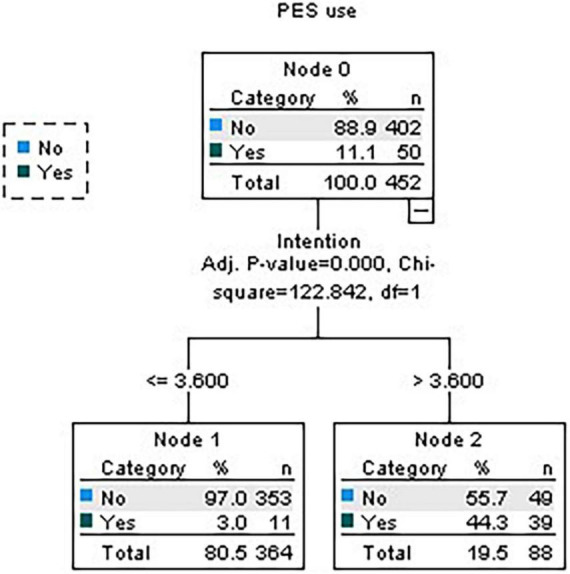
Decision tree for beliefs, subjective norms, attitudes, and intention.

Regarding beliefs, subjective norms, intention, and attitudes, only intention proved to be the most important, with it being found that 97% of individuals who have a negative intention to consume PES (score ≤3.6) do not consume it (Figure 3).

Taking in account that only intentions was significant, the results of multiple linear regression analyzes are presented below. Attitudes, subjective norms, and beliefs proved to be predictors of intention (*p* < 0.0001 for all significant associations), verifying that higher scores on any of the three scales are related to higher intention scores (Table 1).

**TABLE 1:** Multiple linear regression model to identify predictors of intention.

Model^a^	Unstandardized coefficients		*t*	*p*	95% confidence interval for *B*		Collinearity statistics	
	*B*	SE			Lower bound	Upper bound	Tolerance	VIF
(Constant)	−1.000	0.139	−7.196	0.000	−1.273	−0.727		
Attitudes	0.259	0.035	7.438	0.000	0.191	0.328	0.839	1.192
Subjective norms	0.821	0.049	16.610	0.000	0.724	0.918	0.780	1.282
Beliefs	0.302	0.038	7.962	0.000	0.227	0.376	0.784	1.276

^a^Dependent variable: intention.

### 3.4 Global profile

**FIGURE 4:**
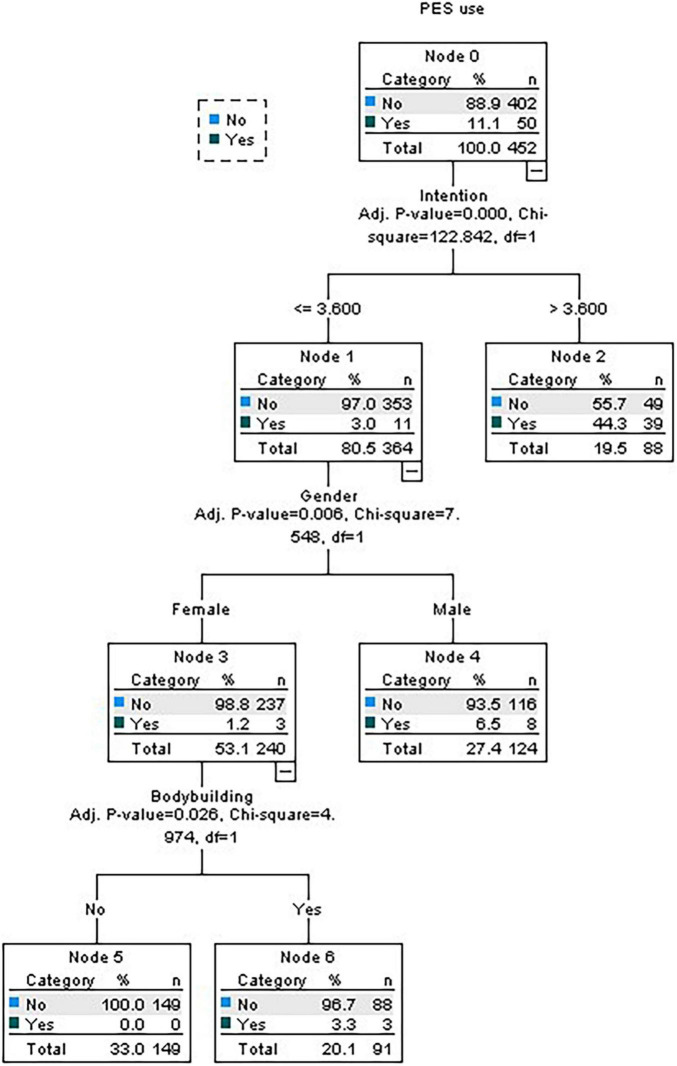
Decision tree for global profile.

Now considering the characteristics that proved to be significant in the previous models, namely age, gender, training frequency, bodybuilding, functional training, and intention, only intention, gender, and bodybuilding proved to be significant. Of those who have a negative intention for PES consumption, were women and do not practice bodybuilding, 100% do not consume PES (Figure 4).

## 4 Discussion

Research on doping and performance enhancement substances, both in competitive sport and in recreational fitness context have mostly focused on the population that consumes this type of products (Wiefferink et al., 2008; Tavares et al., 2020b; García-Grimau et al., 2021). Among these studies several of them have used the TPB to predict this kind of behaviors (Chan et al., 2015a; Kirby et al., 2016; Tavares et al., 2020a). However, as far as we know, no studies have been developed to investigate the protective factors of PES use in fitness context related to non-user population, considering the components of TPB, although a considerable number of studies try to understand why athletes refrain from engaging in PES, on the broad sport context (Leone and Fetro, 2007; Bloodworth and McNamee, 2010; Zenic et al., 2013; Erickson et al., 2015). Therefore, the main purpose of this study was to identify the profile characteristics of the non-use of PES of gym- goers, following the same approach of Tavares et al. (2020a) used to study the determinants to consume PES in the same population. Moreover, the association of gender and type of sport activity with the self-reported behavior of PES was also studied.

More specifically, hypotheses state that the unfavorable evaluation of attitude toward substance consumption, the absence of a subjective norm that emphasizes consumption, and the unfavorable beliefs about the outcomes of PES consumption are associated with the intentions of PES non-use. In line with previous studies (e.g., Lucidi et al., 2004, 2008; Allahverdipour et al., 2012; Chan et al., 2015a), results of the multiple linear regression confirm these hypotheses suggesting that the variation of scores on these variables have a direct and positive relationship with the intention scores. It was also expected that favorable intentions toward PES use were negatively associated with self-reports of PES non-use behavior. In fact, in the current study, when beliefs, subjective norms, intention, and attitudes were considered, only intention was significantly associated to the PES non-use which is supported by the fact that among the non-consuming subjects 97% have a negative intention to use PES. Moreover, when all the components were included in the decision tree model, only intention proved to be significant. This is consistent with the TPB model and results from previous studies investigating PES use (e.g., Lucidi et al., 2004, 2008; Wiefferink et al., 2008) suggesting the need to promote behavior free of PES through educational interventions emphasizing the determinants of intentions of healthy behaviors. For example, taking into consideration the relationship between attitudes and subjective norms with intentions, a main target of educational programs should be the social context of individuals namely the instructors of clubs and exercise centers. Additionally, educational programs aiming at preventing gym-goers’s use of PES should also convey information on the potentially dangerous impact of these substances on health so that negative intentions toward PES develop.

The final hypothesis is also confirmed. It stated that the female practitioners and those who practice non-strength-based activities are more likely to report non-use of PES. Results show the importance of gender as 94.5% of women do not consume PES versus 80% of men, which is in line with other studies (Serpa et al., 2003; Ntoumanis et al., 2014; Sagoe et al., 2015; Backhouse et al., 2016; Nicholls et al., 2017; Tavares et al., 2020a; Collomp et al., 2022). As to the type of activity, 98.1% of the non-consuming subjects do not practice bodybuilding. Additionally, results show that 95.5% of those who have a lower training frequency, and 88% of those who practice functional training, are not PES consumers. These results may be explained by the fact that activities such as cardio fitness, stretching or localized gymnastics are not so connected with results influenced by those substances versus strength-based activities (Wichstrøm, 2006; Tavares et al., 2020b; Mantri et al., 2023). On the other hand, female subjects in the current study are mostly involved in those types of exercising and they may have motivations related to general physical wellbeing instead of over-shaping their muscles with the help of PES. In what concerns the frequency of training per week, it may suggest that physical wellbeing and health goals may be the main purposes to be reached by the lower frequent practitioners. Moreover, in this group there is a significant association with not practicing bodybuilding activities that are very much connected to a certain type of extrinsic social motives facilitating the use of external enhancers (Macho et al., 2021). On the other hand, among the subjects that have a higher frequency per week, most of those who are involved in functional training are PES non-consumers, which suggests that their aim may be being in good physical shape by means of a natural physiological process.

It was also found that the male group under 25 years old emerges significantly as PES non-consumers when compared to older male subjects perhaps because in this period of life subjects feel comfortable with their physical capacities and do not feel the need to enhance them with the help of specific substances. Moreover, intrinsic motives may be stronger than extrinsic ones, which are more strongly related to the use of PES (Ip et al., 2015).

Several aspects were found that are associated to the behaviors of not using PES in the gym-goers population, which are related (i) to cognitive factors analyzed through the TPB framework, (ii) to the subjects’ characteristics and their behavioral approach to the activities, and (iii) to the chosen type of activities. Regarding the cognitive factors, results suggest that intentions related to the PES non-use are strongly associated to this actual behavior. Moreover, individuals who have a negative intention to consume PES do not consume it. As to gender, age and time commitment, female, and younger male gym-users and those with lower frequency of gym activities show the higher percentage of non-use of PES. Regarding the activities, results suggest that functional training and not being involved in bodybuilding are associated to PES non-use.

### 4.1 Practical implications

The findings of the present research study have important implications for current research on the characteristics related to the decision not to use performance-enhancing substances among gym-goers. The analysis of the sample characteristics together with the type of practice gives important information concerning the subjects’ non-use behaviors. Understanding the choices of this specific population concerning not to use PES, could support future intervention strategies to prevent this phenomenon in gymnasium context (Erickson et al., 2015). PES-prevention activities targeting older males and bodybuilding practitioners should form part of a comprehensive multi-systemic PES prevention approach, reinforcing the negative health impact of these substances and healthy alternatives (Horta, 2017). Indeed, medical support and regular health checkups should be promoted among this type of population. Future studies should take in account other psychological constructs such as self-control, resisting social pressure, moral conviction, self-esteem, perfectionist strivings and happiness, as they tend to be associated with PES refusal (Nicholls et al., 2017).

### 4.2 Limitations and future work

Several limitations of this study should be addressed and suggestions for future research considered. First, this study is a cross-sectional survey, which means findings do not inform about the behavior of PES non-use, in those who showed unfavorable beliefs about the outcomes of PES consumption, that is, causal inferences based on the current findings should be avoided. Future studies will benefit from longitudinal designs to enable a precise observation of how unfavorable beliefs about the outcomes of PES consumption in fitness context, could be associated with the absence of PES use behavior, by gym-goers. Second, the non-probabilistic nature of sampling limits the result’s generalization to the wider population of gym-goers. Thirdly, this study was based on self-reporting, which could lend itself to social desirability and response bias. Third, on top of methodological limitations, our study only examined relationships between psychological variables, socio-demographic variables and some types of practice, training frequency, and training time, which limits an understanding of the “whole picture,” concerning the reasons for PES non-use. That is, we do not identify the protective factors that safeguard gym-goers from PES use. According to Chan et al. (2015b), PES use avoidance involve a broad range of behaviors and this behavior could “take place at anytime and anywhere”; hence, methods, such as implicit association tests, might measure this behavior in a more reliable and objective manner. Moreover, a qualitative approach, using data collection techniques like unstructured observation or open interviews, may yield significant insights into the belief systems underpinning gym-goers’ motivation and intentions to not use PES, as it does not entirely define the variables and their values *a priori*, leading new information and knowledge (Lucidi et al., 2016). Finally, it is important to consider the cultural characteristics of the samples because individuals may hold, for example, different values and beliefs grounded on cultural influences or subject to diverse regulatory policies. Nevertheless, studies have utilized samples from a variety of countries (e.g., Iran, Allahverdipour et al., 2012; Netherlands, Wiefferink et al., 2008; Portugal, Tavares et al., 2020a), including Lazuras et al. (2017) cross-cultural study with samples from Cyprus, Greece, UK, Germany, and Italy. More research is needed to explore how cultural factors further influence gym-goers choices concerning PES use or non-use.

## 5 Conclusion

In conclusion, this study shed light on fundamental aspects related to the decision not to use performance-enhancing substances among gym-goers. Findings showed that cognitive factors, particularly negative intentions, play a crucial role in the conscious choice not to use these substances. Furthermore, distinct demographic differences emerged, highlighting a gender disparity, with 94.5% of women and 80% of men opting not to use these substances. Age was also an important characteristic with younger men, especially those under 25, demonstrating significant resistance to PES use, possibly driven by intrinsic motivations and satisfaction with their physical abilities. Regarding activities, those engaged in functional training and not practicing bodybuilding showed a clear preference for not resorting to these substances. Indeed, these younger men may search mostly for the pleasure of the chosen physical practice and not just for its outcome. Moreover, in the younger ages physiological and morphological results are faster and more evident which may reduce the wish to enhance them with PES. Thus, this work provides valuable insights, showing the way for more refined interventions aimed at promoting a culture of health and substance-free fitness practices.

## Data availability statement

The raw data supporting the conclusions of this article will be made available by the authors, without undue reservation.

## Ethics statement

The studies involving humans were approved by the Ethical Committee of the University of Lisbon, Faculty of Human Kinetics. The studies were conducted in accordance with the local legislation and institutional requirements. The participants provided their written informed consent to participate in this study.

## Author contributions

AT: Conceptualization, Data curation, Funding acquisition, Investigation, Methodology, Project administration, Supervision, Validation, Visualization, Writing – original draft, Writing – review & editing. EC: Data curation, Formal analysis, Methodology, Writing – review & editing. PT: Supervision, Writing – original draft, Writing – review & editing. LC: Supervision, Writing – original draft, Writing – review & editing. SS: Conceptualization, Data curation, Methodology, Supervision, Writing – original draft, Writing – review & editing.
