# Supplementary material for: Profile of gym-goers who do not use performance-enhancement substances
Source: Front Psychol. 2024 May 30;15:1357566. doi: 10.3389/fpsyg.2024.1357566 (PMC11169804; doi:10.3389/fpsyg.2024.1357566)
Supplement: Supplementary file 1 [file Data_Sheet_1.PDF]

Appendix A

1. As part of your practice, have you ever taken performance-enhancing substances (PES)*?

- ☐ No
- ☐ Yes. Please indicate which of the substances described below you have used in your physical activity

	Never	1-2 times /week	3-4 times /week	+ 5 times /week
Stimulants (Excite, improve reflexes/concentration capacity and increase aggressiveness. Ex. Amphetamines, ephedrines, cocaine).				
Beta blockers (Relax and prevent hand tremors. Ex. Acebutolol, atenolol, bisoprolol).				
Corticosteroids (Used as anti-inflammatories or analgesics, but also because they provide a certain euphoric effect. Ex. Dexamethasone, prednisolone, triamcinolone).				
Diuretics (Increase urine production and excretion, reduce weight. Ex. Spironolactone, furosemide, thiazides).				
Erythropoietin (EPO) (increases red blood cell production and oxygen transport to muscles).				
Growth hormone (Increases muscle mass without increasing fat mass; Ex. Somatropin).				
Insulins (For non-diabetics, accelerates the burning of sugars, energy production and has an anabolic effect, resulting in performance gains).				
Beta-2-Agonists (They cause bronchodilation and have stimulating and anabolic effects to increase muscles. Ex. Medications against asthma, such as Salbutamol).				
Anabolic steroids (They have an anabolic effect. They make muscles bulkier and give more strength; Ex. Testosterone, nandrolone, stanozolol).				
Substances that reduce side effects (Ex. Tamoxifen, clomiphene).				
Narcotics (Mask the sensation of pain. Ex. Heroin, methadone, morphine).				
Cannabinoids (Relax and improve readiness. Ex. Cannabis, hashish, and marijuana).				
Chorionic Gonadotropin (HCG) (Stimulates the production of testosterone by the testicles. Increases muscle volume and power).				
Other substance				

Indicate which: ____

*Note: PES are considered to be substances taken in non-pharmacological doses (e.g. AAS, amphetamines, diuretics, HCG, etc.), specifically for the purpose of improving physical appearance and increasing performance in physical activity, excluding nutritional supplements.
